# Transcriptome-wide identification of altered RNA m^6^A profiles in cardiac tissue of rats with LPS-induced myocardial injury

**DOI:** 10.3389/fimmu.2023.1122317

**Published:** 2023-05-19

**Authors:** Wei Wang, Tie-Ning Zhang, Ni Yang, Ri Wen, Yu-Jing Wang, Bing-Lun Zhang, Yu-Hang Yang, Chun-Feng Liu

**Affiliations:** Department of Pediatrics, Shengjing Hospital of China Medical University, Shenyang, Liaoning, China

**Keywords:** m^6^A, cardiac, inflammation, apoptosis, endotoxaemia

## Abstract

**Purpose:**

Myocardial injury is a common complication in patients with endotoxaemia/sepsis, especially in children. Moreover, it develops through an unclear pathophysiological mechanism, and effective therapies are lacking. Recently, RNA modification, particularly *N* ^6^-methyladenosine (m^6^A) modification, has been found to be involved in various physiological processes and to play important roles in many diseases. However, the role of m^6^A modification in endotoxaemia/sepsis-induced myocardial injury is still in its infancy. Therefore, we attempted to construct the m^6^A modification map of myocardial injury in a rat model treated by lipopolysaccharide (LPS) and explore the role of m^6^A modification in LPS-induced myocardial injury.

**Method:**

Myocardial injury adolescent rat model was constructed by intraperitoneal injection of LPS. m^6^A RNA Methylation Quantification Kit was used to detect overall level of m^6^A modification in rat cardiac tissue. m^6^A-specific methylated RNA immunoprecipitation followed by high-throughput sequencing (MeRIP-seq) and RNA sequencing (RNA-seq) were conducted to identify the altered m^6^A-modified genes and differentially expressed genes in cardiac tissue of rats treated by LPS and control rats (6 versus. 6). Bioinformatics was used to analyze the functions of differentially m^6^A modified genes, differentially expressed genes, and genes with both differential m^6^A modification and differential expression. qPCR was used to detect expression of m^6^A modification related enzymes.

**Result:**

We found that the overall level of m^6^A modification in cardiac tissue of the LPS group was up-regulated compared with that of the control group. MeRIP-seq and RNA-seq results showed that genes with differential m^6^A modification, genes with differential expression and genes with both differential m^6^A modification and differential expression were closely associated with inflammatory responses and apoptosis. In addition, we found that m^6^A-related enzymes (Mettl16, Rbm15, Fto, Ythdc2 and Hnrnpg) were differentially expressed in the LPS group versus. the control group.

**Conclusion:**

m^6^A modification is involved in the pathogenesis process of LPS-induced myocardial injury, possibly through the regulation of inflammatory response and apoptosis-related pathways. These results provide valuable information regarding the potential pathogenic mechanisms underlying LPS-induced myocardial injury.

## 1 Introduction

Sepsis is a life-threatening organ dysfunction caused by dysregulated host responses to infection (1). Approximately 48.9 million people worldwide are diagnosed with sepsis each year, and sepsis led to ~11 million deaths in 2017 (2). Sepsis is often accompanied by multiple-organ dysfunction, involving conditions such as acute kidney injury, acute lung injury, and myocardial injury. Notably, epidemiological studies have found that the incidence of myocardial injury was as high as 70.2% in children (3), and sepsis-induced myocardial injury was closely related to mortality (4). In addition, endotoxic shock is a subtype of sepsis and is often accompanied by myocardial injury (5, 6). However, the pathophysiological mechanism of endotoxaemia/sepsis-induced myocardial injury is still unclear, and no specific drug can be targeted to treat endotoxaemia/sepsis-induced myocardial injury. Our team has found that myocardial apoptosis, inflammatory disorders (6), mitochondrial dysfunction, oxidative stress (7) and autophagy (8) were involved in the occurrence and development of endotoxaemia/sepsis-induced myocardial injury. Epigenetic modifications have also been implicated (6), but many aspects of the occurrence and development of endotoxaemia/sepsis-induced myocardial injury remain to be discovered.

m^6^A modification is the most frequent internal modification of eukaryotic mRNAs, and such modifications influence diverse fundamental cellular functions, such as pre-mRNA splicing, nuclear transport, stability, translation, and microRNA biogenesis (9). Recently, m^6^A modification has been found to be associated with the occurrence and development of many diseases, such as cancer (9), cardiovascular diseases (10). Moreover, recent studies showed that m^6^A modification played an important regulatory role in mediating inflammation responses, which was to be associated with the occurrence and development of endotoxaemia/sepsis-induced myocardial injury. For example, m^6^A methyltransferase Mettl3 could regulate inflammatory responses through IGF2BP1/HDAC4 dependent manner in LPS-induced cardiomyocytes (11). However, as a widespread RNA modification, m^6^A modification sites and regulatory mechanisms are still worthy of further study.

Here, considering endotoxin can trigger all of the cardinal feature of sepsis on its own and likely is a modulating factor during the syndrome (5), we conducted myocardial injury rat model of LPS-induced endotoxic shock and performed MeRIP-seq and RNA-seq to identify differentially m^6^A-modified RNAs and differentially expressed RNAs involved in LPS-induced myocardial injury. Then, understanding the functions that these differentially m^6^A-modified RNAs may be involved in regulation through bioinformatics. Combined analysis of MeRIP-seq and RNA-seq helped us identify genes that may be regulated by m^6^A at RNA levels. Detection of m^6^A-related enzymes helped us understand which enzymes might play important functions by regulating m^6^A modification.

## 2 Materials and methods

### 2.1 Animals

The study was approved by the Ethics Committee of the Shengjing Hospital of China Medical University (Shenyang, China; approval number 2022PS854K). This study conformed to the relevant ethical standards. The rats used in this study (Beijing HFK Bioscience Co., Ltd.; Beijing, China) had a Wistar genetic background. The rats were adolescent, male, and pathogen-free; weighed approximately 160-180 g; and were housed in cages at 24°C with a 12h alternating light/dark cycle and free access to water and food. An adolescent rat model of endotoxic shock was generated by intraperitoneal (i.p.) injection of LPS, as we described previously (6). Briefly, rats were anesthetized with 20% urethane (1g/kg, i.p.). The left femoral artery was cannulated (Biopac MP150; Biopac Systems, Goleta, CA, USA) to continuously measure the mean arterial pressure (MAP). After the MAP stabilized, LPS (L-2880; Sigma-Aldrich, St. Louis, MO, USA) was administered i.p. (20 mg/kg, 10 mg LPS dissolved in 1 ml of 0.9% saline). 2 hours after LPS injection, MAP began to decline, and the model was considered successful when MAP decreased by 20-25 percent. The control group was injected with an equal volume of 0.9% normal saline solution. The preliminary experiment found that the death rate of rats was relatively high 24 hours after injection of LPS, but could basically be stabilized to 12 hours. In order to obtain the pathological changes in the late stage of LPS-induced myocardial injury, we selected 12 hours after injection of LPS to conduct this study. Twelve hours post-LPS or normal saline injection, euthanasia was performed, and the left ventricle was excised and placed in an RNase-free centrifuge tube, quickly placed in liquid nitrogen, and stored at -80°C. Blood samples were collected from the abdominal aorta at 12h post-LPS or saline administration and centrifuged (3,000 rpm, 10 min) after incubation at room temperature (15°C-25°C) for 15 min. Supernatants were collected and stored at -80°C for enzyme-linked immunosorbent assay (ELISA) analysis. Six pairs of left ventricles with endotoxaemia or controls were selected for MeRIP-seq and RNA-seq, and the remaining samples were saved for validation experiments.

### 2.2 Hematoxylin and eosin staining

Rat heart samples were fixed in 4% paraformaldehyde for 48 h, dehydrated, permeabilized, and embedded in paraffin. Paraffin-embedded tissues were sectioned (0.35 µm) and stained with H&E. Pathological changes were observed microscopically.

### 2.3 Echocardiography

Twelve hours after the LPS injection, echocardiography was performed. A special ultrasound instrument (Vinno Technology, China) for small animals was used to obtain echocardiographic images. Two-dimensional guided M-mode measurements of the left ventricular (LV) internal diameter were obtained from the long-axis view at the level of the papillary muscles. The interventricular septal thickness at diastole (IVSd), left ventricular intra-diameter at diastole (LVIDd), left ventricular end-diastolic posterior wall thickness (LVPWd), interventricular septal thickness at systole (IVSs), left ventricular intra-diameter at systole (LVIDs), left ventricular end-systolic posterior wall thickness (LVPWs), left ventricular end-systolic volume (LVESV), left ventricular end-diastolic volume (LVEDV), left ventricular ejection fraction (LVEF), stroke volume (SV), left ventricular fractional shortening (LVFS), heart rate (HR) and cardiac output (CO) were recorded.

### 2.4 ELISA-based analysis of rat serum

Cardiac troponin I (c-TNI), creatine kinase myocardial band (CK-MB) and interleukin (IL)-6 levels in blood supernatants from both groups were determined *via* ELISA analysis. IL-6 ELISA kits were purchased from Wanlei Biological Company (Shenyang, China), whereas CK-MB and c-TNI ELISA kits were purchased from Elabscience Biotechnology Co., Ltd. (Wuhan, China). The biomarker concentrations in each sample were estimated based on optical density values at 450 nm and a standard curve.

### 2.5 Measurement of total m^6^A levels

Total RNA was isolated from heart tissue using the TRIzol Reagent (Invitrogen, Life Technologies, Carlsbad, CA, USA). The RNA quantity and purity of each sample were determined using Nanodrop 2000 (Thermo Scientific, USA). m^6^A RNA Methylation Quantification Kit (ab185912, Abcam, Shanghai, China) was used to detect the total m^6^A levels in 200ng RNA by measuring absorbance at 450nm.

### 2.6 MeRIP-seq and RNA-seq analyses

RNA extraction and purity measurement are the same as 2.5. RNA-integrity numbers were assessed using a Bioanalyzer 2100 (Agilent, CA, USA) and exceeded 7.0, as confirmed by denaturing agarose-gel electrophoresis. Poly (A) RNA was purified from 50μg total RNA using Dynabeads Oligo (dT)25-61005 (Thermo Fisher, CA, USA) and two rounds of purification. Then, the poly(A) RNA was fragmented using a Magnesium RNA Fragmentation Module (NEB, USA) at 86°C for 7min. The RNA fragments were incubated for 2h at 4°C with an m^6^A-specific antibody (Synaptic Systems, Germany) in IP buffer (50mM Tris-HCl, 750mM NaCl, and 0.5% Igepal CA-630). Both the input samples (without IP) and the m^6^A-IP samples were used to construct RNA-seq libraries with the NEBNext Ultra RNA Library Prep Kit for Illumina. Finally, we performed 2×150bp paired-end sequencing (PE150) on an Illumina Novaseq 6000 instrument (LC-Bio Technology Co., Ltd., Hangzhou, China).

### 2.7 Bioinformatics analysis

Fastp software (12) (default parameters) was used to remove reads containing adaptors, low-quality bases, and undetermined bases. The quality of the IP and input sequences was verified using FastQC and RseQC. Filtered high-quality data were mapped to the *Rattus norvegicus* genome (Ensemble release 101) using HISAT2 (13). Peak calling and differential-peak analysis were performed using the exomePeak2 package (14), and peaks were annotated using ANNOVAR. The peaks in each group with a fold change (FC) of ≥2 was considered significant. HOMER (15) was used for *de novo* motif finding, followed by motif localization with respect to the peak summit. Then, StringTie (16) was used to determine the expression levels of all mRNAs from the input libraries using the following relationship: (total exon fragments/mapped reads [millions] × exon length [kB]).

### 2.8 Protein–protein-interaction network

Genes with differential expression or m^6^A modification sites were imported into the STRING database (17), and the interaction relationships between the imported genes were determined. A PPI network was constructed by importing the data into Cytoscape 3.8.2 (18)and analyzing the data using a network analyzer. The genes imported with combined interaction scores of >0.4 were selected to construct the PPI network.

### 2.9 MeRIP assays

MeRIP assays were performed using the m^6^A RNA Enrichment Kit (Epigentek, USA). Target m^6^A-containing fragments were immunoprecipitated using a bead-bound m^6^A capture antibody, and RNA sequences at both ends of the m^6^A-containing regions were cleaved using Cleavage Enzyme Mix. The enriched RNA was released, purified, and eluted. Quantitative reverse transcriptase-PCR (qRT-PCR) analysis was performed following MeRIP to quantify changes in target-gene m^6^A modification. Primers (**Table S1**) were synthesized by Sangon Biotech Co., Ltd. (Shanghai, China).

### 2.10 Real-time RT-PCR analysis

RNA was reverse transcribed to complementary DNA using the PrimeScript™ RT Reagent Kit with gDNA Eraser (Takara Bio, Being, China), with a reaction time of 15min at 37°C, followed by denaturation for 5s at 85°C. A 7500 Real-Time PCR System (Applied Biosystems, USA) was used for PCR. The primers used are listed in **Table S2**.

### 2.11 Western blot

Frozen ventricular tissue samples were homogenized in RIPA buffer (Beyotime Biotechnology, China) containing protein enzyme inhibitors, and protein concentration was quantified using a BCA Protein Assay Kit (Beyotime Biotechnology, China). The lysates were separated on 8–15% SDS-PAGE gels and then electro-transferred to 0.45μm polyvinylidene difluoride membranes (Millipore, USA). After blocking with 5% nonfat milk in Tris-buffered saline at room temperature for 2h, the membranes were incubated for overnight at 4°C with primary antibodies. The primary antibody included IL-6 (Wanlei, China), IL-1β (Affinity, China), IL-18 (Affinity, China) and Tubulin (Affinity, China). After washing three times for 10min per wash, the membranes were incubated with an HRP-conjugated secondary antibody for 2h at room temperature. Subsequently, the blots were imaged using a Bio-Rad imaging system (Bio-Rad, USA) and protein expression levels were determined using ImageJ software.

### 2.12 Statistical analysis

All data were obtained from three or more independent experiments and are presented as mean ± standard deviation. Statistical analyses were conducted using GraphPad Prism (version 8.0). Student’s t-test for unpaired data was used to compare two groups. Statistical significance was set at P < 0.05. Peaks and transcripts with |FC| ≥1.5 and P < 0.05 between control group and LPS group were considered differential peaks and transcripts.

## 3 Results

### 3.1 Myocardial injury induced by LPS was linked to changes in global m^6^A modification level

**Figure 1:**
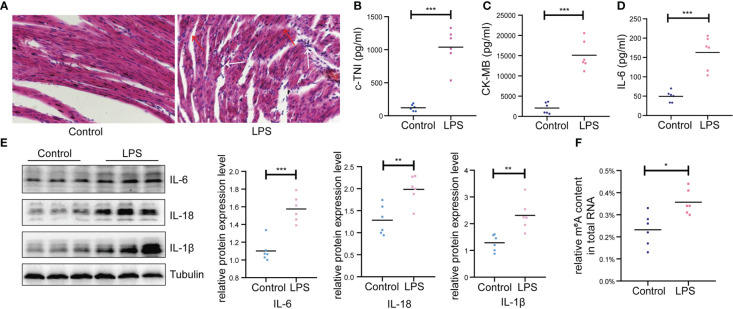
Basic characteristics of animal models between LPS group and control group. **(A)** Gross morphology of cardiac tissue stained with HE (200×) (n=6 in each group); Red arrow, disordered cardiac cells; White arrow, infiltration of inflammatory cells. **(B, C)** Serum cardiac enzyme c-TNI and CK-MB **(D)** Serum inflammatory biomarker IL-6 were detected by ELISA (n=6 in each group). **(E)** Protein expression levels of inflammatory indicators (IL-6, IL-18, IL-1β) were detected by western blot (n=6 in each group). **(F)** Quantification of m^6^A levels in cardiac tissue of LPS group and control group (n=6 in each group). HE, hematoxylin and eosin; c-TNI, cardiac troponin I; CK-MB, creatine kinase MB form; *p < 0.05 compared to the control; **p < 0.01 compared to the control; ***p < 0.001 compared to the control.

In this study, we conducted a rat model of endotoxic shock by intraperitoneal injection of LPS. The MAP in rats decreased by 25-30% 2h after LPS administration (**Supplementary Figure 1**). Firstly, we assessed cardiac function using echocardiograph. As shown in **Table 1**, the IVSd, LVIDd, LVIDs, LVEDV, LVESV, SV, HR and CO were deceased significantly in the LPS group while the LVPWd and LVSs were increased significantly in the LPS group. However, no difference in LVPWs, EF and FS were observed between two groups. Then, we detected the pathological changes in the cardiac tissue founding that myocardial cells showed greater disorder and inflammatory cell infiltration in the LPS group than in the control group (H&E staining; **Figure 1A**). Strikingly, serum c-TNI and CK-MB (markers of injury to cardiomyocytes) were significantly elevated in the LPS group (**Figure 1B, C**), as was the serum inflammatory biomarker, IL-6 (**Figure 1D**). We also analyzed the protein expression levels of inflammatory cytokines (IL-6, IL-18, IL-1β) in the heart using western blot. The protein expression levels of IL-6, IL-18, and IL-1β were upregulated in the LPS group compared to the control group (**Figure 1E**), suggesting inflammatory injury of cardiac tissue in the rat model induced by LPS.

**Table 1:** The echocardiographic data of rats in groups.

	Control	LPS
IVSd (mm)	379.300 ± 52.520	303.400 ± 33.230*
LVIDd (mm)	6.395 ± 0.247	3.563 ± 0.433***
LVPWd (mm)	1.313 ± 0.228	2.423 ± 0.301***
IVSs (mm)	2.863 ± 0.197	3.328± 0.339*
LVIDs (mm)	1.615 ± 0.240	0.937 ± 0.209***
LVPWs (mm)	3.337 ± 0.239	3.522 ± 0.300
LVEDV (ml)	0.603 ± 0.063	0.118 ± 0.042***
LVESV (ml)	0.012 ± 0.004	0.002 ± 0.004**
LVEF (%)	98.000 ± 0.859	97.890 ± 0.912
SV (ml)	0.592 ± 0.063	0.117 ± 0.043***
LVFS (%)	74.760 ± 3.484	73.800 ± 4.191
HR (time/min)	379.300 ± 52.520	303.400 ± 33.230*
CO (L/min)	0.223 ± 0.037	0.036 ± 0.015***

n=6 per group; * p < 0.05 compared to the control; **p < 0.01 compared to the control; ***p < 0.001 compared to the control; IVSd, interventricular septal thickness at diastole; LVIDd, left ventricular intra-diameter at diastole; LVPWd, left ventricular end-diastolic posterior wall thickness; IVSs, interventricular septal thickness at systole; LVIDs, left ventricular intra-diameter at systole; LVPWs, left ventricular end-systolic posterior wall thickness; LVESV, left ventricular end-systolic volume; LVEDV, left ventricular end-diastolic volume; LVEF, left ventricular ejection fraction; SV, stroke volume; LVFS, left ventricular fractional shortening; HR, heart rate; CO, cardiac output.

In addition, higher total m^6^A modification levels were found in the LPS group than that in the control group (**Figure 1F**). Thus, we performed transcriptome-wide MeRIP-seq and RNA-Seq to generate an m^6^A modification map in cardiac tissue of rats with LPS-induced myocardial injury.

### 3.2 Differential m^6^A modification patterns in cardiac tissue of rats treated by LPS and control rats

**Figure 2:**
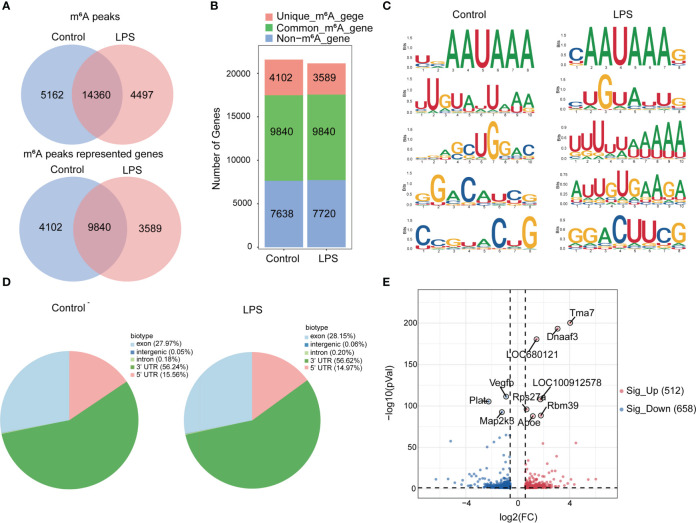
Transcriptome-wide MeRIP-seq and analysis of m^6^A peaks. **(A)** Overlap of m^6^A peaks in the LPS group and the control group. Numbers of LPS-unique, control-unique, and common m^6^A peaks are shown as Venn diagram as well as m^6^A peaks representing genes in the two groups. **(B)** Summary of genes with m^6^A modification identified in MeRIP-seq. **(C)** Top 5 m^6^A modification motifs enriched from all identified m^6^A peaks in the two groups. **(D)** Proportion of m^6^A peaks distributed in the indicated regions in the control group and the LPS group. **(E)** Volcano map: identification of 512 hyper-m^6^A modification and 658 hypo-m^6^A modification peaks in the LPS group compared to the control group (|FC|>=1.5, P*<*0.05). n=6 in each group; FC, fold change.

Next, we compared the m^6^A peaks between LPS group and control group. We identified 14,360 common m^6^A peaks, representing 9,840 genes in both groups using exomePeak2 (**Figure 2A**). In addition, 4,497 unique peaks and 3,589 unique genes appeared in the LPS group, whereas 5,162 unique peaks and 4,102 unique genes appeared in the control group (**Figure 2B**). Chromosome 1 showed the largest number of m^6^A modification sites with 2,272 and 2,355 m^6^A peaks in the LPS group and the control group, respectively, followed by chromosome 10 with 1,613 and 1,662 m^6^A peaks in the LPS group and the control group, respectively (**Supplementary Figure 2A**). Most genes had one to three m^6^A modification sites (**Supplementary Figure 2B**).

In addition, we identified the top five consensus motifs for the m^6^A peaks in the LPS group and the control group (**Figure 2C**). RRACH motif was identified in both groups, where R = G or A; A = m^6^A, and H = U, A, or C, as described (19). The m^6^A peaks were predominantly distributed in coding sequences and the 3’ untranslated region (UTR) (**Supplementary Figure 2C**). Finally, m^6^A peaks were analyzed in whole transcriptome data and divided into exon, intergenic, intron, 3’UTR, 5’UTR regions, based on their location in RNA transcripts. The peaks were mainly distributed in 3’UTR, exon and 5’UTR and different distribution patterns were observed between the two groups. The LPS group showed slightly more m^6^A peaks in the exon (27.97% versus. 28.15%) and 3′UTR (56.24% versus. 56.62%) and slightly less m^6^A peaks in the 5′UTR (15.56% versus. 14.97%) than the control group (**Figure 2D**).

Furthermore, we found 14,360 m^6^A peaks in the LPS group and the control group, 5,740 of which were differentially modification (P < 0.05). Among them, 512 hypermethylated and 658 hypomethylated m^6^A modification sites (|FC| ≥ 1.5, P < 0.05) were found in the LPS group versus. the control group. The 10 genes with the most significant differences were identified (**Figure 2E**). **Table 2** showed the top 20 differentially upregulated and downregulated m^6^A peaks.

**Table 2:** Top 20 differentially up- and down-regulated m^6^A peaks according to the fold change in the LPS group compared to the control group.

Chr	Peak_length	Strand	DiffMod Log2FC	P-value	Annotation	Transcript ID	Gene Name
chr1	551	–	6.02	0.00	exonic	ENSRNOT00000044195	LOC100911951
chr7	1067	–	5.44	0.00	exonic	ENSRNOT00000086062	Col2a1
chr1	466	+	4.48	0.00	UTR3	ENSRNOT00000028222	Polr2i
chr10	2933	+	4.36	0.00	exonic	ENSRNOT00000089404	AC117889
chr2	1476	–	4.11	0.00	UTR3	ENSRNOT00000009813	Fgb
chr8	4687	–	4.04	0.00	UTR3	ENSRNOT00000075819	Tma7
chr5	401	+	3.86	0.00	UTR3	ENSRNOT00000037547	MGC94199
chr1	101	+	3.60	0.00	exonic	ENSRNOT00000017948	Mrgprf
chr9	376	–	3.50	0.00	UTR3	ENSRNOT00000016237	LOC100365697
chr9	449	+	3.30	0.00	UTR5	ENSRNOT00000051841	Atn1
chr3	201	+	3.17	0.00	UTR3	ENSRNOT00000022265	RGD1561517
chr1	727	+	3.07	0.00	UTR3	ENSRNOT00000058900	Dnaaf3
chr1	3661	+	3.00	0.00	exonic	ENSRNOT00000016883	Cyp2e1
chr18	226	+	2.95	0.00	exonic	ENSRNOT00000027015	Pcdhga7
chr8	9941	+	2.89	0.00	UTR3	ENSRNOT00000047613	Faim
chr14	376	+	2.88	0.00	UTR3	ENSRNOT00000033437	Gpr75
chr17	2440	–	2.78	0.00	exonic	ENSRNOT00000010836	LOC100912163
chr10	373	+	2.64	0.00	UTR3	ENSRNOT00000091610	Gspt1
chr13	376	+	2.62	0.00	UTR3	ENSRNOT00000004104	Gpr161
chr13	376	–	2.61	0.00	UTR3	ENSRNOT00000004204	LOC100911825
chr20	101	–	-6.31	0.00	exonic	ENSRNOT00000073474	RT1-DMb
chr20	426	–	-5.37	0.00	UTR3	ENSRNOT00000086240	RT1-DMb
chr7	2975	–	-5.17	0.00	UTR5	ENSRNOT00000021523	Calcoco1
chr1	319	+	-4.85	0.00	exonic	ENSRNOT00000064178	LOC100911727
chr12	101	+	-4.33	0.00	exonic	ENSRNOT00000001479	Fbxl18
chr11	251	–	-4.25	0.00	exonic	ENSRNOT00000029842	LOC100911374
chr1	101	+	-3.82	0.00	UTR5	ENSRNOT00000074301	Zfp260
chr20	3286	–	-3.72	0.00	exonic	ENSRNOT00000073474	RT1-DMb
chr20	126	+	-3.68	0.00	UTR3	ENSRNOT00000000468	Slc35f1
chr1	7568	–	-3.59	0.00	exonic	ENSRNOT00000017718	Lnpep
chr12	5279	+	-3.56	0.00	exonic	ENSRNOT00000070868	Gatc
chr15	301	+	-3.36	0.00	UTR3	ENSRNOT00000074912	LOC108348161
chr20	226	–	-3.32	0.00	UTR3	ENSRNOT00000070886	RT1-DMa
chr10	176	–	-3.20	0.00	UTR3	ENSRNOT00000073706	LOC100912585
chr1	126	–	-3.08	0.00	UTR3	ENSRNOT00000075570	Itprip
chr10	611	–	-3.07	0.00	exonic	ENSRNOT00000013281	Exoc7
chr14	401	–	-2.93	0.00	UTR3	ENSRNOT00000075415	Pigg
chr18	1176	+	-2.89	0.00	UTR3	ENSRNOT00000073388	LOC100910979
chr9	6739	–	-2.71	0.00	UTR5	ENSRNOT00000019848	Tmbim1
chr11	101	–	-2.60	0.01	exonic	ENSRNOT00000002713	Tmprss2

### 3.3 Systematic functional analysis of genes with differential m^6^A modification

To determine the possible functions of the differentially m^6^A-methylated genes involved in regulation, we performed Gene Ontology (GO) (20), Kyoto Encyclopedia of Genes and Genomes (KEGG) (21), and PPI network analyses.

**Figure 3:**
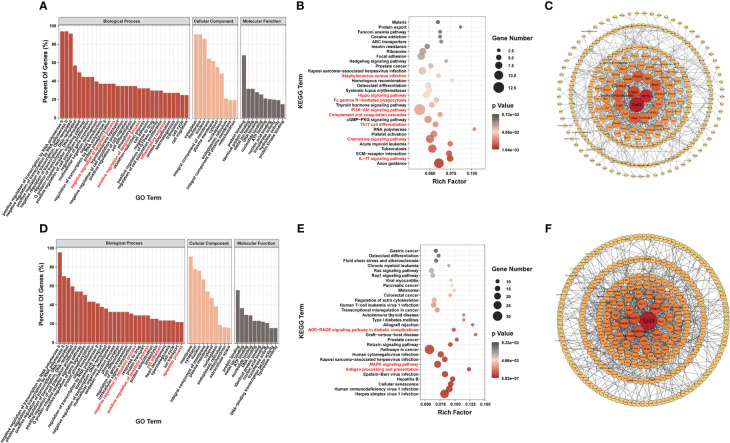
Systematic functional analysis of genes with differentially m^6^A modification. **(A–C)** Systematic functional analysis for genes with up-regulated m^6^A modification; **(A)** Major gene ontology terms significantly enriched; **(B)** Top 30 significantly enriched pathways; **(C)** PPI network. **(D–F)** Systematic functional analysis for genes with down-regulated m^6^A modification; **(D)** Major gene ontology terms significantly enriched; **(E)** Top 30 significantly enriched pathways; **(F)** PPI network.

Most genes with differential m^6^A modification were related to apoptosis and inflammatory responses by GO analysis (**Figures 3A, D**). Furthermore, KEGG pathway analysis of genes with differentially m^6^A modification revealed enrichment for terms related to (i) inflammatory responses, such as *Staphylococcus aureus* infection, Fc gamma R-mediated phagocytosis, complement and coagulation cascades, Th17 cell differentiation, chemokine signaling pathway, IL17 signaling pathway and antigen processing and presentation; (ii) important mediators of apoptosis, such as Hippo signaling pathway, AGE-RAGE signaling pathway in diabetic complications, MAPK signaling pathway and PI3K/Akt signaling pathway (**Figures 3B, E**).

We then performed PPI-network analysis of genes with differential m^6^A modification using Cytoscape (18). Stat3, Hif1a, and Creb1 were the most central genes with upregulated m^6^A modification, whereas Tp53, Mapk11, and Hsp90aa1 were the most central genes with downregulated m^6^A modification (**Figures 3C, F**). These central proteins are particularly associated with inflammatory responses and apoptosis.

### 3.4 mRNA-expression differences in cardiac tissue of rats treated by LPS and control rats

**Figure 4:**
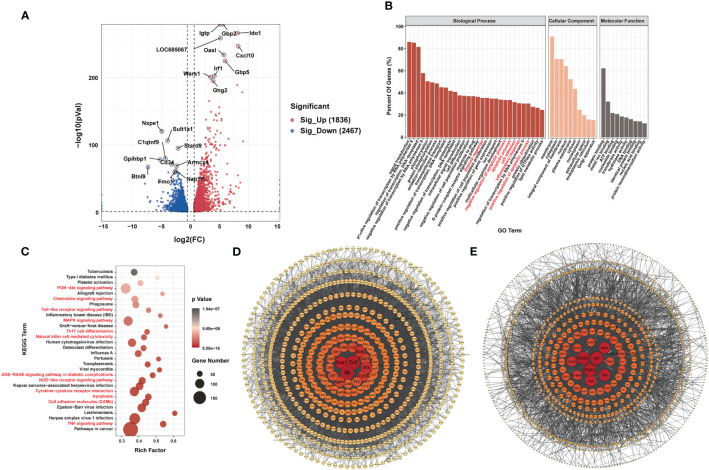
Differences in mRNA Expression in cardiac tissue of the LPS group and the control group. **(A)** Volcano map of differential expression genes (|FC|>=1.5, P<0.05). **(B)** Major gene ontology terms significantly enriched. **(C)** Top 30 significantly enriched pathways. **(D)** PPI network for up-regulated genes. **(E)** PPI network for down-regulated genes. n = 6 in each group.

RNA-seq data showed that 1,836 genes were significantly upregulated and that 2,467 genes were significantly downregulated in the LPS group compared to the control group (|FC| ≥1.5, P < 0.05). **Figure 4A** shows the 10 most upregulated and downregulated genes. In addition, principal component analysis showed that samples from the LPS group and control group clustered separately within each group (**Supplementary Figure 3A**). The heatmap of Pearson correlation coefficient matrix also revealed good intragroup correlations in both groups (**Supplementary Figure 3B**). Moreover, GO analysis suggested that differentially expressed genes were associated with inflammatory responses and apoptosis (**Figure 4B**). KEGG pathway analysis revealed enrichment for terms related to (i) inflammatory responses terms, such as the chemokine signaling pathway, Toll-like receptor signaling pathway, Th17 cell differentiation, natural killer cell-mediated cytotoxicity, NOD-like receptor signaling pathway, cytokine-cytokine receptor interaction and TNF signaling pathway; (ii) apoptosis terms such as PI3K-Akt signaling pathway and MAPK signaling pathway (**Figure 4C**). Furthermore, PPI-network analysis demonstrated that the central proteins were Stat1, Tnf, and IL-6 among the upregulated genes (which are critical for inflammatory responses), and Erbb3, Igf1, and Pik3r3 among the downregulated genes (which are vital for involvement of the PI3k-Akt signaling pathway) (**Figures 4D, E**).

### 3.5 Conjoint analysis of MeRIP-seq and RNA-seq data

**Figure 5:**
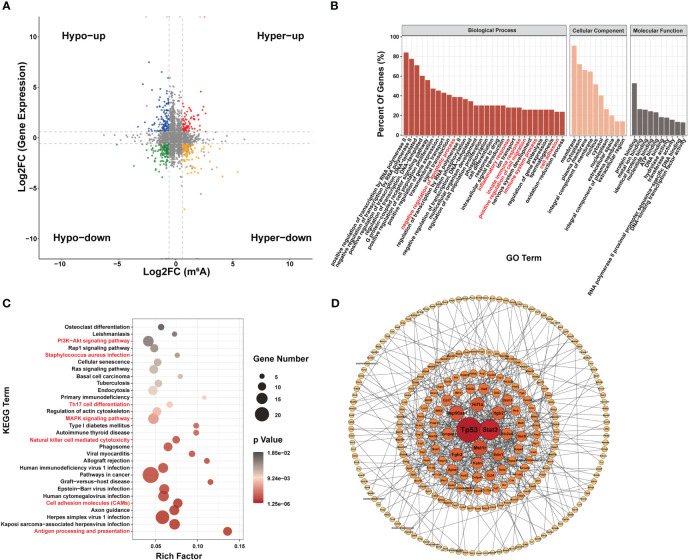
Conjoint analysis of MeRIP-seq and RNA-seq data. **(A)** Four-quadrant diagram of m^6^A modification and RNA expression, divided into hypo m^6^A modification with gene up regulation (Hypo-up), hyper m^6^A modification with gene up regulation (Hyper-up), hypo m^6^A modification with gene down regulation (Hypo-down) and hyper m^6^A modification with gene down regulation (Hyper-down). **(B–D)** Function analysis of gene with both differential m^6^A modification and differential expression; **(B)** Major gene ontology terms significantly enriched; **(C)** Top 30 significantly enriched pathways; **(D)** PPI network.

Seventy-two genes with m^6^A hypermodification and 103 genes with m^6^A hypomodification were upregulated. In addition, 137 genes with m^6^A hypermodification and 133 genes with m^6^A hypomodification were downregulated. Notably, 4 upregulated genes had both m^6^A upregulated and downregulated modification at different sites, and 9 downregulated genes had both m^6^A upregulated and downregulated modification sites (**Figure 5A**).

GO analysis suggested that genes with both differential m^6^A modification and differential expression were related to inflammatory responses and apoptosis (**Figure 5B**). KEGG terms related to (i) certain immune pathways, such as *Staphylococcus aureus* sepsis, Th17 cell differentiation, natural killer cell-mediated cytotoxicity, cell adhesion molecules and antigen processing and presentation and (ii) several pathways involved in apoptosis, such as PI3K-Akt signaling pathway and MAPK signaling pathway, were significantly enriched (**Figure 5C**). In addition, Tp53, stat3, Mst1r, and Hsp90aa1 were the most central proteins in the PPI network associated with immune response and apoptosis (**Figure 5D**).

**Figure 6:**
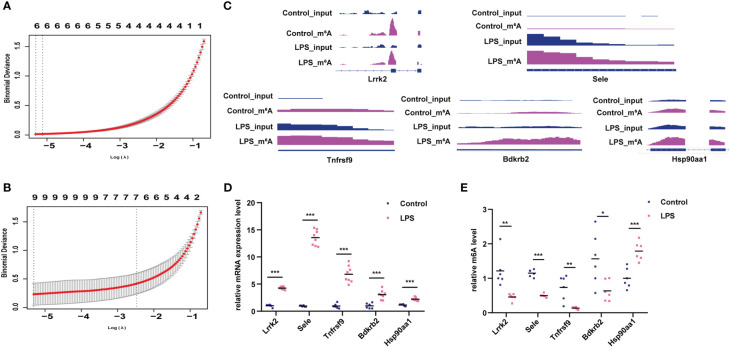
Verification of genes with differential m^6^A modification and differential expression. **(A)** Lasso analysis for genes with hyper-m^6^A modification. **(B)** Lasso analysis for genes with hypo-m^6^A modification. **(C)** m^6^A peak and gene expression visualization using IGV in the LPS group and control group. **(D, E)** Verification of m^6^A modification and gene expression levels of Lrrk2, Sele, Tnfrsf9, Bdkrb2, Hsp90aa1 in the LPS group compared to the control group. n = 6 in each group; *p < 0.05 compared to the control; **p < 0.01 compared to the control. ***p < 0.001 compared to control.

Lasso screening identified Utrn, Stat3, Stard9, Pcdh12, LOC108348047, and Dnmt3a as key m^6^A-hypermodified genes (**Figure 6A**), whereas Tnfrsf9, Sele, Pcdh12, Myrip, Gng2, Gng12, Bdkrb2, Batf, and Agrn were identified as key m^6^A-hypomodified genes in the LPS group compared to the control group (**Figure 6B**). Furthermore, we analyzed the m^6^A modification sites with the Integrative Genomics Viewer (IGV) (22) (**Figure 6C**), verified the m^6^A modification and gene expression levels of leucine-rich repeat kinase 2 (Lrrk2), selectin E (Sele), TNF receptor superfamily member 9 (Tnfrsf9), bradykinin receptor B2 (Bdkrb2), and heat shock protein 90 alpha family class A member 1 (Hsp90aa1) in the LPS group and the control group (**Figures 6D, E**), which showed that they had the same expression tendencies, consistent with our sequencing data.

### 3.6 Changes in m^6^A-related enzymes in cardiac tissue of rats treated by LPS and control rats

**Figure 7:**
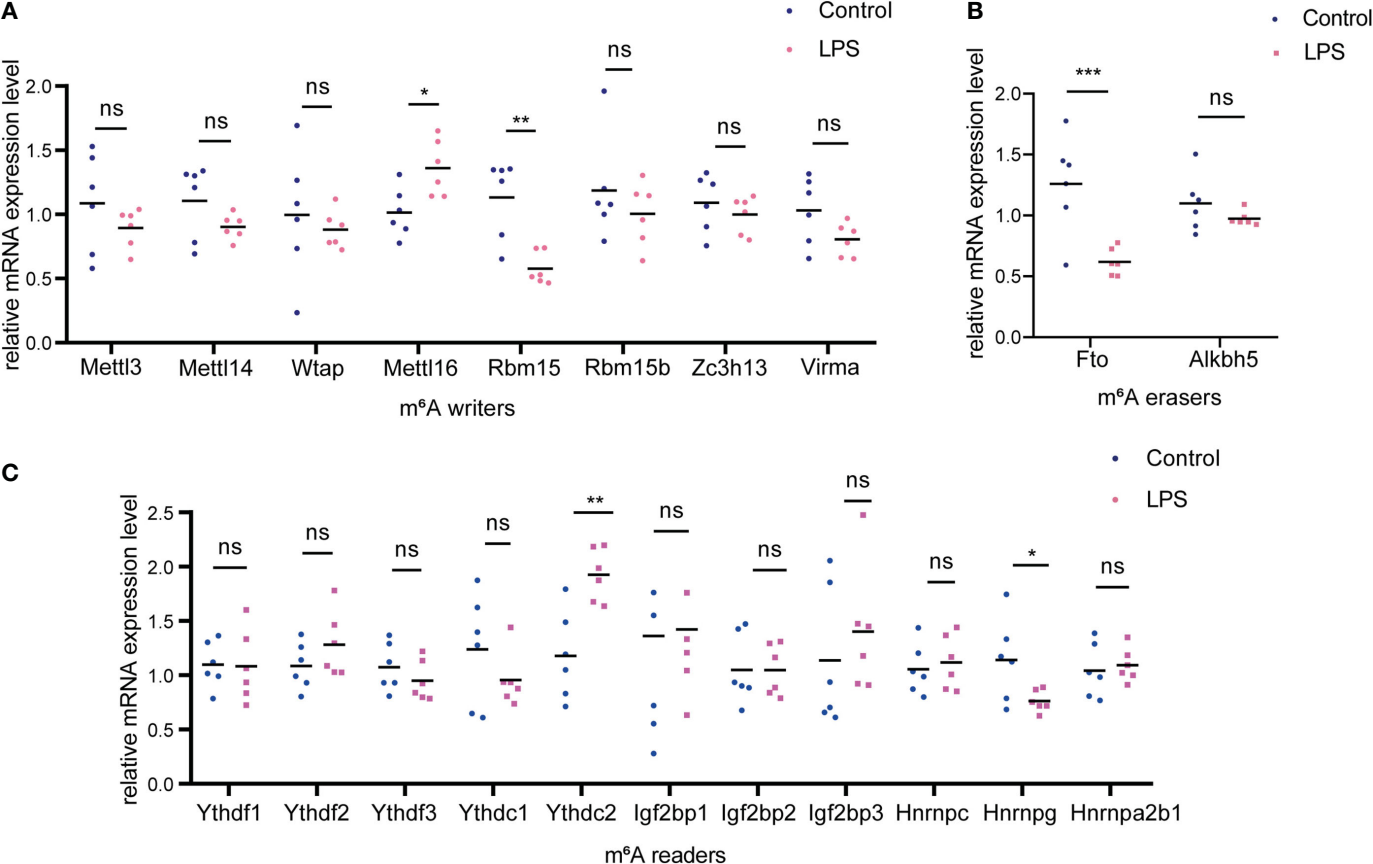
Changes of m^6^A-related enzymes in cardiac tissue of the LPS group compared to the control group. **(A)** mRNA expression levels of m^6^A methyltransferases, also named as “m^6^A writers” in the LPS group and the control group. **(B)** mRNA expression levels of m^6^A demethylases, also named as “erasers”; **(C)** mRNA expression levels of m^6^A recognition factors, called as “readers”. n=6 in each group; *p < 0.05 compared to the control; **p < 0.01 compared to the control. ***p < 0.001 compared to control. ns, no significance.

We examined the expression of m^6^A methyltransferase (“writers”), including Mettl3, Mettl14, Wtap, Mettl16, Rbm15, Rbm15b, Zc3h13 and Virma and found that Mettl16 was upregulated whereas Rbm15 was significantly downregulated in the LPS group versus. the control group (**Figure 7A**). In addition, among the m^6^A demethylases (“erasers”) studied (Fto and Alkbh5), Fto was significantly downregulated in the LPS group (**Figure 7B**). When studying m^6^A-recognition factors (“readers”), we discovered that the YTH domain family protein, Ythdc2, was significantly upregulated in the LPS group versus. the control group and that heterogeneous nuclear ribonucleoprotein G (Hnrnpg) was downregulated (**Figure 7C**).

## 4 Discussion

The main features of endotoxaemia/sepsis-induced myocardial injury are ventricular dilatation, reduced ventricular contractility, and right and left ventricular dysfunction with a reduced response to volume infusion (23). However, no targeted treatments are available for this condition. Additionally, the molecular mechanisms underlying endotoxaemia/sepsis-induced myocardial injury remain unclear. Thus, it is necessary to explore the potential pathophysiological mechanisms underlying endotoxaemia/sepsis-induced myocardial injury. m^6^A modification is a widespread mRNA modification in eukaryotes that participates in post-transcriptional gene regulation (9). Regulators of m^6^A modification include m^6^A writers, erasers, and readers. m^6^A writers include Mettl3, Mettl14, Wtap, Rbm15/15b, Virma (Kiaa1429), and Zc3h13, which function together with m^6^A methyltransferase or cooperatively regulate m^6^A modification. Notably, dynamic m^6^A methylation can be reversed by nuclear m^6^A erasers, including Fto and Alkbh5 (24). In addition, m^6^A readers can regulate mRNA splicing, nuclear export, decay/degradation, translation, and stability by passing the m^6^A signal (9). The dysregulation of m^6^A modification and m^6^A-related enzymes have been found to be associated with cardiac diseases, such as myocardial infarction (10), heart failure (19) and cardiac hypertrophy (25), but the role of m^6^A modification in endotoxaemia/sepsis-induced myocardial injury is relatively unexplored.

In this study, we conducted the rat model of LPS-induced endotoxic shock. Strikingly, we did not find changes in ejection fraction and shortening fraction of echocardiography results. We speculated that the possible reasons were: 1. Myocardial injury was not serious enough; 2. Ejection fraction and shortening fraction were affected by reduced afterload (26, 27), resulting from vascular paralysis existed in our model. Therefore, the ejection fraction and shortening fraction may remain unchanged even with reduced cardiac systolic function. Some studies have proposed that the diagnosis of endotoxaemia/sepsis-induced myocardial injury based on EF values may underestimate its incidence and lead to increased mortality (26). In addition, the reductions in LVID, LVEDV, cardiac output, and stroke volume in this study may also be secondary to reduced preload. To investigate whether cardiac systolic dysfunction existed in the rat model of LPS induced endotoxic shock, we detected cardiac systolic function by an invasive hemodynamic study in previous studies (6, 7, 28). The MAP began decreasing 2 h after LPS injection, accompanied by significant decreases in the heart rate, peak rate of left ventricular pressure rise, and left ventricular peak rate of pressure decay, as well as a prolonged relaxation time constant. These studies showed that our model existed reduced cardiac systolic function and was stable. In this study, although we did not conduct invasive hemodynamics study, we did examine the decreased MAP as an indicator of endotoxic shock.

Moreover, as highly sensitive and specific markers of myocardial damage, clinical studies have proved that troponin (cTn) including c-TNI and c-TNT were correlated with a greater degree of left ventricular dysfunction, illness severity, and mortality of sepsis patients (23, 29, 30). CK-MB was also biochemical markers of myocyte necrosis (31). We found that the markers of myocardial injury (c-TNI and CK-MB) of serum were up-regulated significantly, indicating the presence of some extent of myocardial injury in the LPS group. In addition, inflammatory markers including IL-6, IL-18 and IL1β of cardiac tissue were also up-regulated significantly, indicating inflammatory injury in the LPS group. Furthermore, we have found increased cardiomyocyte apoptosis with LPS challenge by TUNEL staining in previous studies (6, 32). Therefore, there was myocardial injury in the LPS-induced endotoxic shock rats constructed in this study.

We performed MeRIP-seq and RNA-seq in cardiac tissue of rats treated by LPS and control rats. Bioinformatics analysis revealed that differentially expressed RNAs were mainly concentrated in inflammatory responses and apoptotic pathways. For example, the Hippo signaling pathway, AGE-RAGE signaling pathway in diabetic complications, MAPK signaling pathway and PI3K/Akt signaling pathway are closely associated with apoptosis (33–36). Our team have found infiltration of inflammatory cells and cardiomyocytes apoptosis existed in the cardiac tissue of rats with LPS-induced myocardial injury in previous study (7, 32). Other studies have also shown that inflammation and apoptosis are important pathophysiological processes of LPS-induced myocardial injury (37, 38). For example, TNFα, IL-6, IL1β and other inflammatory factors can directly or indirectly cause myocardial injury (39, 40). Chinese herbal preparations may alleviate LPS-induced myocardial injury by inhibiting myocardial apoptosis (41). These studies suggested the important role of apoptosis and inflammation in LPS-induced myocardial injury. Strikingly, we identified 512 hypermethylated and 658 hypomethylated m^6^A peaks in the LPS group compared to the control group by MeRIP-seq. GO and KEGG analyses showed that genes with differential m^6^A modification were also significantly associated with inflammatory responses terms and apoptosis terms, indicating potential important role in LPS-induced myocardial injury. In addition, Dubey et al. demonstrated that increased m^6^A-RNA methylation and Fto suppression was associated with myocardial inflammation and dysfunction during endotoxaemia in mice (42) and Shen et al. reported that Mettl3 knockdown repressed the inflammatory damage of LPS-induced cardiomyocytes by regulating m^6^A modification on HDAC4 mRNA (11). These studies indicated that m^6^A modification was involved in molecular regulation of inflammation in LPS-induced myocardial injury. In this study, we explored the differential m^6^A modification sites in cardiac tissue of rats with LPS-induced myocardial injury and control rats using deep sequencing, providing more potential regulation mechanisms and therapeutic targets. For example, we found that Stat3, Hif1a and Creb1 were the most interacting genes among the up-regulated m^6^A modified genes, while Tp53, Mapk11 and Hsp90aa1 were the most interacting genes among the down-regulated m^6^A modified genes by PPI network analysis. Tp53, Creb1, Mapk11 and Hif1a have been reported to be closely related to apoptosis (43–46), while Stat3 and Hsp90aa1 are classical molecules of inflammatory response (47, 48). Our sequencing results found that these genes were modified by m^6^A and what role does m^6^A play in these genes deserve further investigation in LPS-induced myocardial injury. Moreover, other apoptotic and inflammatory pathways in the MeRIP-seq results are also worthy of further exploration, including PI3K-Akt signaling pathway, Hippo signal pathway, AGE-RAGE signaling pathway in diabetes complications, MAPK signaling pathway, etc (33–36). These studies and results suggested that m^6^A modification may play an important role in LPS-induced myocardial injury through regulation of apoptosis and inflammatory response.

Notably, m^6^A methylation participates in the post-transcriptional regulation of genes and may regulate RNA-expression levels. Therefore, we performed combined analysis of MeRIP-seq and RNA-seq to identify potential genes that may be regulated by m^6^A modification at RNA level. Enrichment results of genes with both differential m^6^A modification and differential expression showed that inflammatory responses and apoptosis were significantly enriched. Moreover, Lasso analysis was used to identify key genes that may be regulated by m^6^A modification (49). These genes may play a more important role in LPS-induced myocardial injury and deserve further attention. Furthermore, we performed MeRIP-qPCR and qPCR to verify the m^6^A modification and RNA expression levels of Lrrk2, Sele, Tnfrsf9, Bdkrb2, and Hsp90aa1 in the LPS group and the control group. Lrrk2 is closely associated with the MAPK pathway and participates in many inflammatory diseases (50, 51). Recently, Liu et al. reported that Lrrk2 deficiency protected against cardiac remodeling under pressure overload (52). Sele, as an adhesion molecular, was reported to be involved in occurrence and development of various inflammatory diseases (53). Tnfrsf9 is involved in T cell and natural killer cell activation and cytokine production and plays a critical role in LPS-induced septic shock (54). Bdkrb2, a receptor for bradykinin which act as mediators of pain and inflammation, can activate a phosphatidylinositol-calcium second messenger system (55). Hsp90aa1, an inducible molecular chaperone that functions as a homodimer, can bind LPS and mediates LPS-induced inflammatory response, including Tnf secretion by monocytes (48). In addition, GO analysis revealed that Tnfrsf9, Bdkrb2, and Hsp90aa1 were significantly enriched in the apoptosis pathway. These genes with both differential expression and differential m^6^A modification provide a new idea for molecular regulation mechanism of LPS-induced myocardial injury. Furthermore, we found Mettl16, Rbm15, Fto, Ythdc2, and Hnrnpg were differentially expressed which may participate in regulation genes with differentially m^6^A modification in cardiac tissue of rats with LPS-induced myocardial injury compared to control rats, providing a new perspective for further exploring the regulation mechanism of m^6^A modification.

## 5 Conclusion

In this study, we established m^6^A modification map in cardiac tissue of rats with LPS-induced myocardial injury and found that genes with differential expression and differential m^6^A modification were closely related to inflammation and apoptosis, which enriched the pathophysiological processes of LPS-induced myocardial injury and provided new ideas for more researchers. This study also has some limitations, such as the lack of deeper mechanistic research, the lack of exploration of m^6^A modification changes at early stage of endotoxaemia and MeRIP-seq including only mRNA but not non-coding RNAs. More research is needed to explore these aspects in the future.

## Data availability statement

The datasets presented in this study can be found in online repositories. The names of the repository/repositories and accession number(s) can be found below: PRJNA911952 (SRA).

## Ethics statement

The animal study was reviewed and approved by the Ethics Committee of the Shengjing Hospital of China Medical University (Shenyang, China; approval number 2022PS854K).

## Author contributions

C-FL performed study concept and design. WW performed development of methodology and writing. T-NZ and NY performed review and revision of the paper. RW, Y-JW, B-LZ, and HY provided acquisition, analysis and interpretation of data, and statistical analysis. All authors read and approved the final paper.

## Supplementary material

Supplementary Figure 1 The MAP changes in rats after LPS administration. The rats were divided into control group and LPS group, and the changes of blood pressure over time were measured by femoral artery intubation, including 0h, 2h, 6h, and 12h after injection of LPS or normal saline. n=6 in each group; ***p < 0.001 compared to control.

Supplementary Figure 2 Basic characteristics of m^6^A peaks in the LPS group and the control group. **(A)** Number of m^6^A peaks in each chromosome in both groups. **(B)** Number of m^6^A peaks in each gene in both groups. **(C)** Distribution of m^6^A peaks in 5’UTR, CDS and 3’UTR in both groups.

Supplementary Figure 3 Correlation analysis of RNA-seq in the LPS group and the control group. **(A)** Principal component analysis (PCA) of RNA-seq in both groups. **(B)** Pearson correlation heatmap of RNA-seq in in both groups.
