# Supplementary material for: Transcriptome-wide identification of altered RNA m6A profiles in cardiac tissue of rats with LPS-induced myocardial injury
Source: Front Immunol. 2023 May 19;14:1122317. doi: 10.3389/fimmu.2023.1122317 (PMC10237353; doi:10.3389/fimmu.2023.1122317)
Supplement: Supplementary file 1 [file Table_1.docx]

| Genes | Primer F | Primer R |
| --- | --- | --- |
| Lrrk2 | AGCATCCTAAGGGCATCATTTCACG | CGCCATGTAGTTCTTAGGGAATCGC |
| Sele | TGTTCCTGCACAAAGTGTCCCAAC | CCACCAACCCCAATCAGCAAGG |
| Tnfrsf9 | CCAGAGGAAGAAGAAGGAGGAGGAG | GGGTCTTAGTGCTTCTCGGTTTCC |
| Bdkrb2 | CTGAGGACCTCTATCTCGGTGGA | GATGGCTTGTGTTCACTGCTTGTTC |
| Hsp90aa1 | ACAAAGCCGATCTGGACCAGAAATC | TGTTCTTCCCAGTCGTTGGTTAAGC |

**Table S1. Primers for MeRIP-qPCR.**
