# Supplementary material for: Transcriptome-wide identification of altered RNA m6A profiles in cardiac tissue of rats with LPS-induced myocardial injury
Source: Front Immunol. 2023 May 19;14:1122317. doi: 10.3389/fimmu.2023.1122317 (PMC10237353; doi:10.3389/fimmu.2023.1122317)
Supplement: Supplementary file 2 [file Table_2.docx]

| Genes | Primer F | Primer R |
| --- | --- | --- |
| Mettl3 | AGGTTCGTTCCACCAGTCAC | ATCCAGTTGGGCTGCACATT |
| Mettl14 | GACACCCAAGTTTGATGTGATTC | CCACGTCCAGCACTTCTCGT |
| Wtap | AAGTTATGGCACGGGATGAGT | CCTGCTGTTGCTGCTTTAGTTTT |
| Mettl16 | GGAAGCCAAGGGTGTAAACTCTCG | CCTCTGCCATGATCTCTGTGATGC |
| Rbm15 | CCGTCACTGCTTATGAGCCACTG | CAAGATGCCGTCCTCCACTTTCC |
| Rbm15b | TTCTGGCGAGTCTAGCAGGAGTC | GAGGCAGCAGGAGCAAAGTGTC |
| Virma | GTGCTAAGAGGCTGGTACAACTGTC | TGGAGGAGGAGGTGGAGGAGAG |
| Zc3h13 | CGCACTCCTAGTCCACCTCCTC | TCCCGTCCTCTGTCCTTTCCATC |
| Fto | GACGAGAACTTGGTGGACAGG | ATCCAGCATGAAATAGCAGTCTC |
| Alkbh5 | GTTCTTATGTTCTTGGCTTTCCTC | CCCATTATCTCTACTGGCTACTCTG |
| Ythdf1 | CCCTGTCCTGGAGAAACTGAAAGC | GTACTTGATGGAGCGGTGGATGTC |
| Ythdf2 | TTGCCTCCACCTCCACCACAG | CCCATTATGACCGAACCCACTGC |
| Ythdf3 | GCAGCCTCAGCCACCACAAC | TCCGAGGAGCTACCCAACGATTC |
| Ythtdc1 | TCTGAACAGGGCAACAACACTGAG | CCTCCTCCTCCTCCGCATCTTC |
| Ythdc2 | GACCAGCCAGACTGACAAGTAACG | GCGGTAGTTCTATCTTCCATCTCACTG |
| Igf2bp1 | CCAAGACGGCAGACGAAGTTCC | AAGTTCCGCCCTTCCTTTCCAATG |
| Igf2bp2 | CGCCAGATGAGAACGAGGAAGTG | CCTGCTGCTTCACCTGCTGTAC |
| Igf2bp3 | AACGGCAGAGGATTCGGAAACTTC | TCACAGCTCTCCACCACTCCATAC |
| Hnrnpg | AAGGATGCTGCCAGAGACATGAATG | TGCTTCTTGGAGGTGGAGGTAGTC |
| Hnrnpc | TTCACGATTGCAGTAGTCGCTTCC | AGGGAAAACACACGCCTCTAATGC |
| Hnrnpa2b1 | AGCAACCTTCTAACTACGGTCCAATG | CTCAATATCGGCTCCTTCCACCATAG |

**Table S2. Primers for qPCR of m6A-related enzymes.**
