# Supplementary figures and images for: Transcriptome-wide identification of altered RNA m6A profiles in cardiac tissue of rats with LPS-induced myocardial injury
Source: Front Immunol. 2023 May 19;14:1122317. doi: 10.3389/fimmu.2023.1122317 (PMC10237353; doi:10.3389/fimmu.2023.1122317)

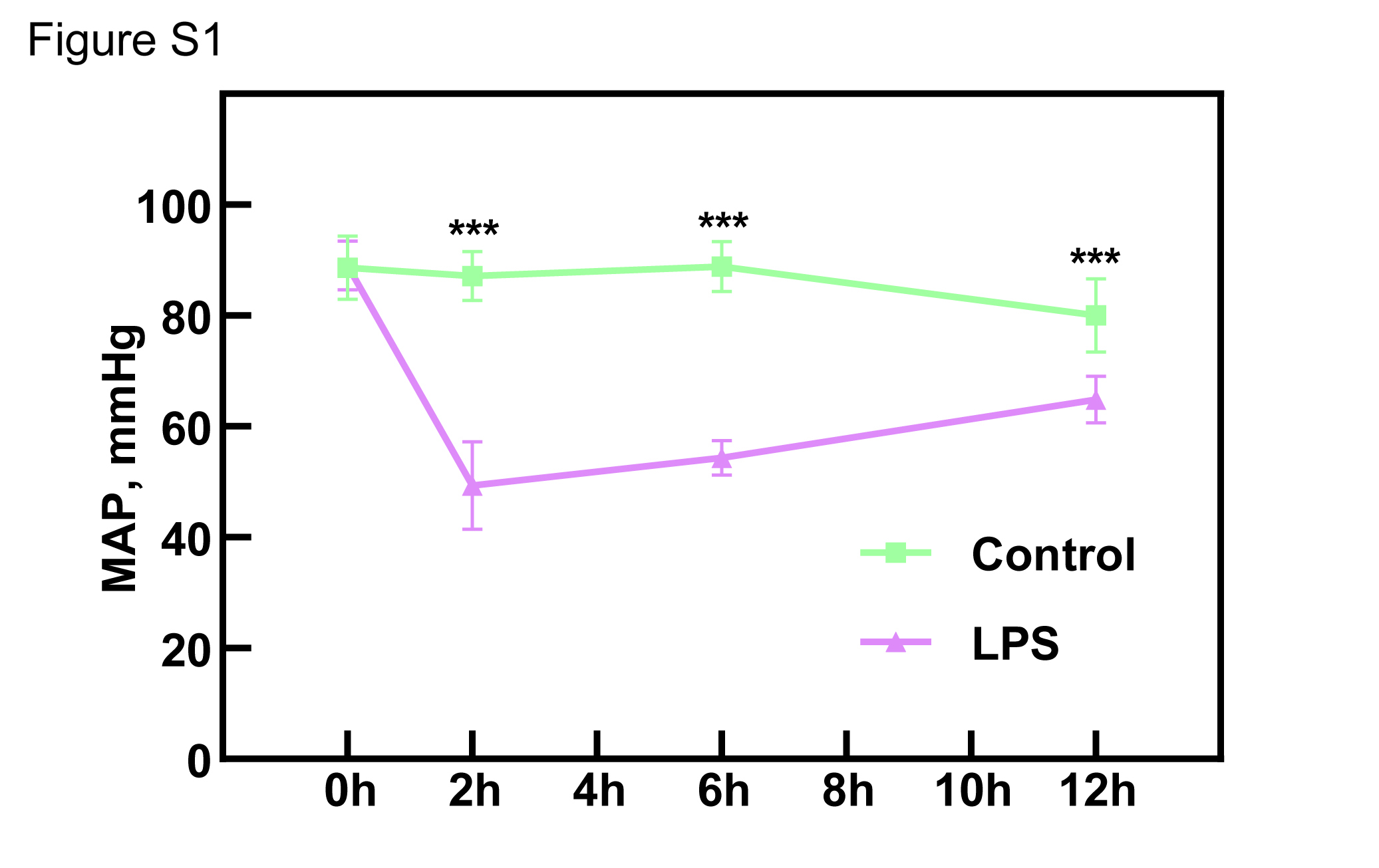

Supplement: Supplementary Figure 1 — The MAP changes in rats after LPS administration. The rats were divided into control group and LPS group, and the changes of blood pressure over time were measured by femoral artery intubation, including 0h, 2h, 6h, and 12h after injection of LPS or normal saline. n=6 in each group; ***p < 0.001 compared to control. [file Image_1.jpeg]

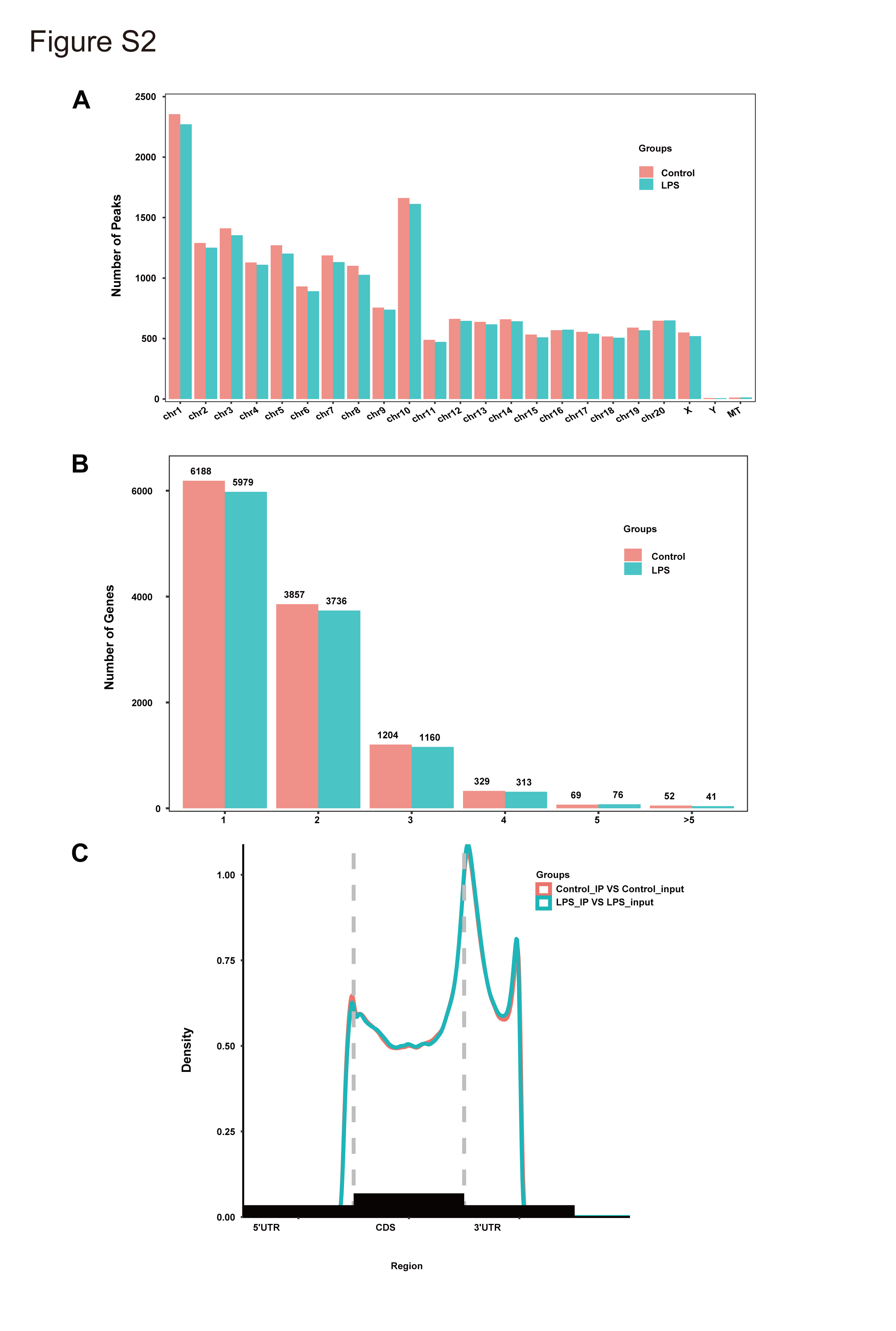

Supplement: Supplementary Figure 2 — Basic characteristics of m6A peaks in the LPS group and the control group. (A) Number of m6A peaks in each chromosome in both groups. (B) Number of m6A peaks in each gene in both groups. (C) Distribution of m6A peaks in 5’UTR, CDS and 3’UTR in both groups. [file Image_2.jpeg]

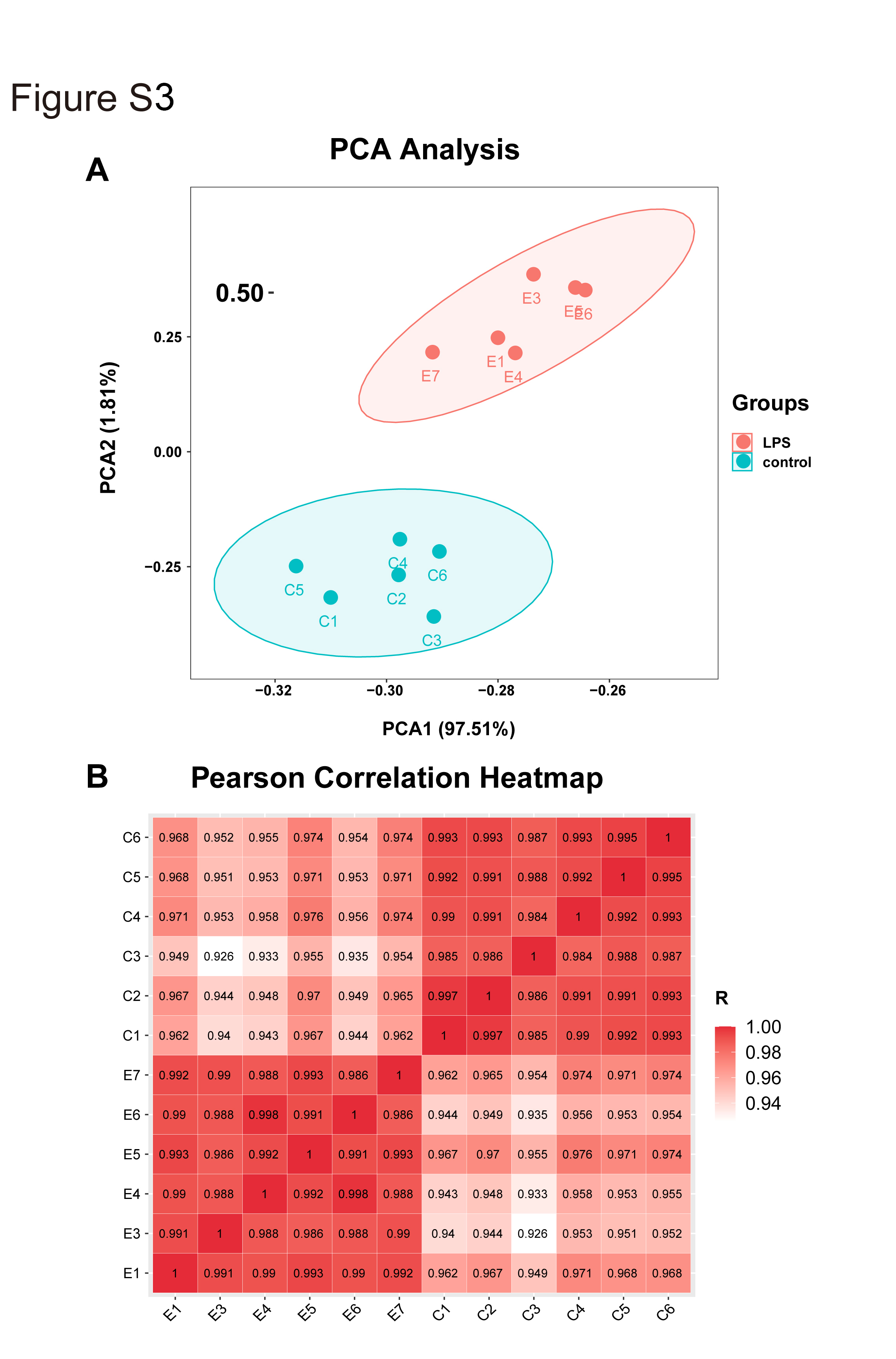

Supplement: Supplementary Figure 3 — Correlation analysis of RNA-seq in the LPS group and the control group. (A) Principal component analysis (PCA) of RNA-seq in both groups. (B) Pearson correlation heatmap of RNA-seq in in both groups. [file Image_3.jpeg]
